# Mechanism of transformation in *Mycobacteria* using a novel shockwave assisted technique driven by in-situ generated oxyhydrogen

**DOI:** 10.1038/s41598-017-08542-5

**Published:** 2017-08-17

**Authors:** Akshay Datey, Janardhanraj Subburaj, Jagadeesh Gopalan, Dipshikha Chakravortty

**Affiliations:** 1 Department of Aerospace Engineering, Indian Institute of Science, Bangalore, India; 2 Department of Microbiology and Cell Biology, Indian Institute of Science, Bangalore, India; 3 Centre for Biosystems Science and Engineering, Indian Institute of Science, Bangalore, India

## Abstract

We present a novel method for shockwave-assisted bacterial transformation using a miniature oxyhydrogen detonation-driven shock tube. We have obtained transformation efficiencies of about 1.28 × 10^6^, 1.7 × 10^6^, 5 × 10^6^, 1 × 10^5^, 1 × 10^5^ and 2 × 10^5^ transformants/µg of DNA for *Escherichia coli*, *Salmonella* Typhimurum, *Pseudomonas aeruginosa, Mycobacterium smegmatis*, *Mycobacterium tuberculosis* (Mtb) and *Helicobacter pylori* respectively using this method which are significantly higher than those obtained using conventional methods. Mtb is the most difficult bacteria to be transformed and hence their genetic modification is hampered due to their poor transformation efficiency. Experimental results show that longer steady time duration of the shockwave results in higher transformation efficiencies. Measurements of Young’s modulus and rigidity of cell wall give a good understanding of the transformation mechanism and these results have been validated computationally. We describe the development of a novel shockwave device for efficient bacterial transformation in complex bacteria along with experimental evidence for understanding the transformation mechanism.

## Introduction

Transferring DNA/RNA to the cells is an important requisite for basic as well as applied biotechnology and molecular biology studies. Transformation, transduction and conjugation are the natural ways of DNA uptake known in bacteria. Interestingly, not all the bacterial species are naturally competent for transformation unlike *Bacillus subtilis, Haemophilus influenzae*, and *Streptococcus pneumoniae* ^1, 2^. Transduction is a host specific process of nucleic acid transfer whereas conjugation is limited by the requirement of physical contact between the donor and the recipient bacteria under the influence of a bacterium harboring a helper plasmid^1, 3^. Owing to these limitations, inducing artificial competence has been widely adapted to transform a wide range of hosts from bacteria to complex mammalian and plant cells. Liposome or polymeric based transfection agents, viral vectors and electroporation are the majorly used methods to transform living cells. Electroporation is a mechanical method where electrical discharge makes the cells permissive for the uptake of nucleic acids^2^. *Mycobacteria* are the most difficult to transform because of a waxy thick layer of mycolic acid present in its cell wall. It was observed that when the cells were exposed to shock waves in the presence of nucleic acids, they could take up the DNA/RNA^4^. Many dry particle delivery systems have been developed using high pressure gases to accelerate particles for plant transformation^5, 6^. Therefore, shockwaves prove their importance and role in inducing cell competence. Although, these methods have been successful in inducing competence, their mechanisms have still not been understood.

Shockwaves are a means by which the sudden release of energy in nature is dissipated to its environment at speeds greater than the local speed of sound^7^. There are non-linear waves which cause an instantaneous increase in pressure, temperature and density of the medium they propagate through^7^. Cell transformation using shockwaves is a method that has caught the attention of researchers worldwide due to its potential to obtain high transformation efficiencies even in organisms with a thick cell wall. Previously an underwater shockwave generator has been shown to successfully transform bacteria^4^. Nanothermite based system to perform cell transformation have also been reported^8^. PDMS (Polydimethylsiloxane) membrane was used to isolate the combustion products from the living cells. A novel shock wave-induced transfer of DNA into bacteria using dual-pulse (tandem) shockwaves with 50-fold higher transformation efficiency by enhancing cavitation is also reported^9^. Although the shockwave based cell transformation systems are effective, their efficiencies are inferior to the conventional methods. A promising method is reported which uses detonation transmission tubing for bacterial transformation. This method employs an explosive coated polymer tube known as the NONEL tube to generate micro-shockwaves^10^. The transformation efficiencies obtained using the NONEL tube system is at par with the conventional methods used. This system also has added advantages of being simple, less costly and portable. The NONEL tube system has an upper limit on the strength of the shockwave that is generated. Hence, the application is limited only to a few strains of bacteria^11^. In the present study, a 6mm miniature shock tube driven by *in situ* generated oxyhydrogen is used^12^. High transformation efficiencies were obtained in bacterial strains of *Escherichia coli, Salmonella* Typhimurium*, Pseudomonas aeruginosa, Mycobacterium smegmatis, Mycobacterium tuberculosis and Helicobacter pylori*. The ability of the proposed device to produce shockwaves of required strength in a safe, clean and repeatable manner opens new opportunities for shockwave-assisted biomedical research^12^. Different shock-wave sources namely argon fluoride excimer laser, ruby laser, and shock tube were used to perform bacterial transformation^13^. It was shown that the impulse of the shock wave (i.e., the pressure integrated over time) plays a more dominant role in creating cell permeability rather than the peak pressure of the shockwave. This device demonstrates potential for delivery of exogenous DNA through cell wall of simple as well as complex bacterial strains. Bacterial cell wall has a complex structure which prevents the bacteria from environmental challenges and helps maintain its internal homeostasis. The mechanics of the cell wall have been studied in the recent past which includes measurements of properties like rigidity and Young’s modulus^14^. The porosity and permeability of the cell wall play a vital role in the sustenance of a bacterial cell and its ability to take up foreign DNA. In this article, we report the development of a novel shockwave generator, its application for bacterial transformation and the mechanical changes caused by shockwave exposure and its importance in the mechanism of efficient transformation.

## Results

### Design of the oxyhydrogen detonation-driven miniature shock tube for bacterial transformation

**Figure 1:**
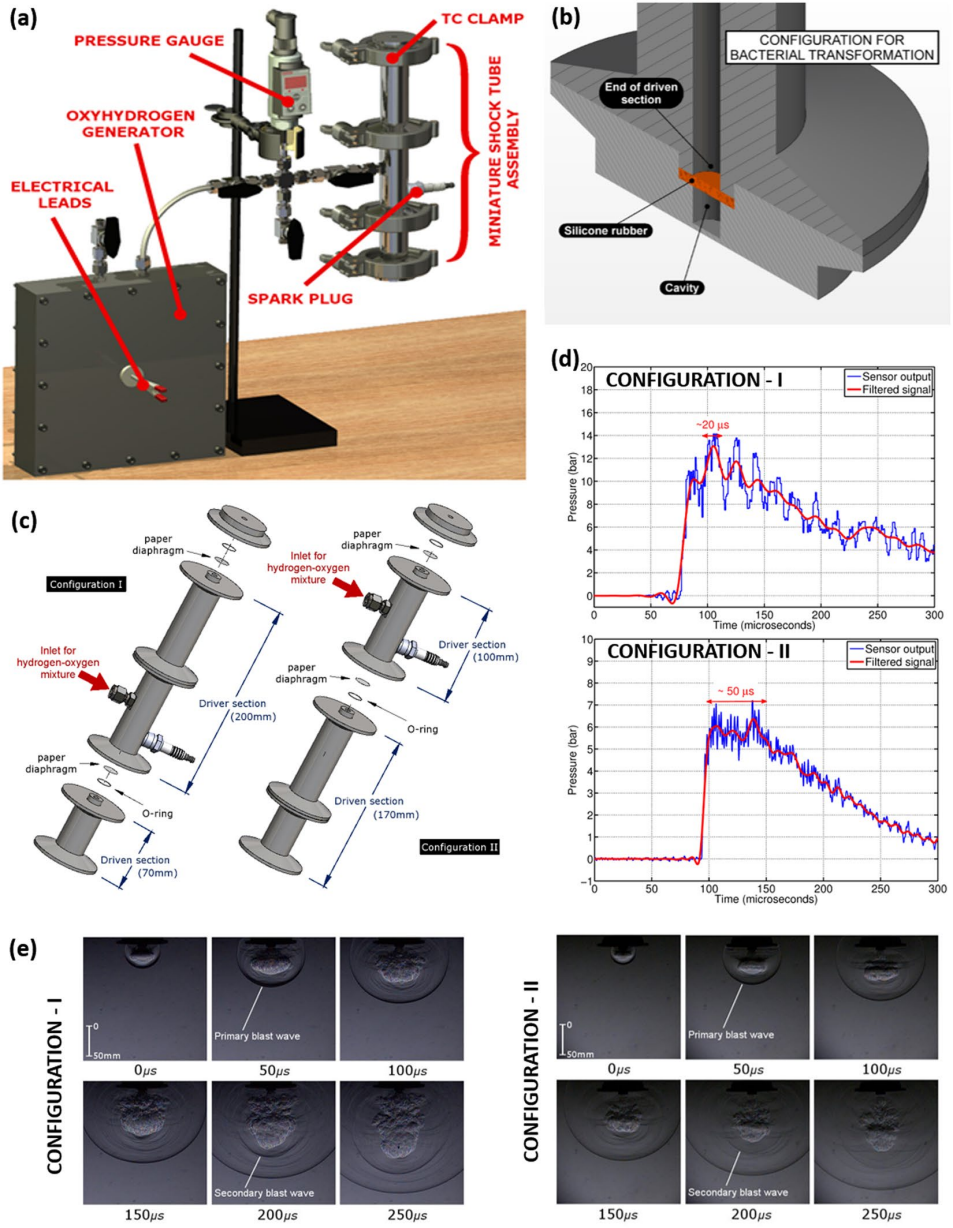
The oxyhydrogen-driven miniature shockwave device for bacterial transformation. **(a)** Schematic of the oxyhydrogen-driven miniature shockwave device for bacterial transformation, **(b)** Cross-sectional view of the end of the shock tube showing the cavity for biological sample, **(c)** Shock tube configurations used for the study. Different lengths of driver and driven section are depicted, **(d)** Pressure signal recorded for the two configurations using a sensor flush mounted to the wall of the driven section placed 12 mm from the end of the shock tube, **(e)** High speed Schlieren images of the blast wave recorded from the open end of the shock tube operated using the two configurations.

An oxyhydrogen detonation-driven miniature shock tube assembly to generate shockwaves of required strength and duration has been reported^12^. A similar experimental setup with slight modifications has been used for the present work (Fig. 1a). The device comprises of two main components - an oxyhydrogen generator and a miniature shock tube assembly. The oxyhydrogen generator produces the required amount of stoichiometric mixture of hydrogen and oxygen gases through alkaline electrolysis. A miniature shock tube assembly with an internal diameter of 6mm is used. The oxyhydrogen mixture is filled in the driver section of the shock tube (this is termed as initial fill pressure of oxyhydrogen henceforth) and a spark plug, placed close to the diaphragm station between the driver and the driven section, is used to ignite the mixture to produce a backward facing detonation front (Supplementary Fig. S1a). The high pressure and temperature behind the detonation front causes the instantaneous rupture of the diaphragm between the driver and driven section and produces a strong shockwave in the driven section of the shock tube. Tracing paper (95 GSM) is used as diaphragm in the shock tube which can be replaced by a quick opening solenoid valve at a later stage. A tri-clover clamp is used between the different sections of the shock tube to facilitate quicker and easier changing of diaphragm after each experiment. The biological sample is accommodated in a stainless steel sterile cavity of diameter 6mm and depth 5 mm (Fig. 1b). The optimization of the dimension of the cavity has already been reported^5^. In a previous study, a brass foil was suggested as a viable option for energy transfer from the shock wave to the biological sample and also to avoid contamination of the bacteria by the products of detonation^10^. However, brass foil needs to be replacement after every experiment and also it absorbs most of the incident shockwave energy before transmitting it to the biological sample. Therefore, in the present work, we have used a silicone rubber membrane to separate the cavity housing the biological sample from the shock tube. Silicone rubber is a biocompatible material which has a good tensile strength and is resistant to temperatures of up to 300 °C^15^. These properties make it ideal for our application, as there is no need for frequent replacement and the energy transfer is much better as compared to using the brass foil.

### Effect of steady time of the shock wave on bacterial transformation

It has been reported that the impulse of the shockwave (i.e. pressure integrated over time) rather than the peak pressure plays a more vital role in changing the permeability of the membrane to achieve better molecular delivery^13^. The impulse of the shockwave is a function of the steady time duration as well as the amplitude of the shock wave. It is calculated by isolating the first peak in the pressure vs time plot. Impulse per unit area (I) can be represented by the formula,

$$I=\,{\int }_{0}^{T}P(t)dt$$

where ‘T’ represents the steady time of the shock wave, ‘P(t)’ is the pressure experienced by the sample as a function of time. The amplitude, rise time, steady time and fall time of a typical shockwave signal is shown (Supplementary Fig. S1b). The area under the curve represented by the shaded region in the figure gives the impulse per unit area generated. The effect of steady time and amplitude of the shockwave can be easily verified using the present experimental setup as changing the length of the driver and driven sections of the shock tube is one simple way of varying these parameters^16, 17^. Different combinations of driver and driven lengths were chosen to arrive at two configurations of shock tube (Fig. 1c). A tri-clover assembly used to clamp the different sections of the shock tube makes diaphragm changing and length variation in the shock tube effortless. For the same initial fill pressure in the driver section (3 bar of oxyhydrogen mixture), configuration I generates an incident shock wave with a high-pressure amplitude and low steady time as compared to configuration II, which generates an incident shock wave with a low-pressure amplitude and high steady time duration (Fig. 1d). Figure 1e shows the time resolved high-speed schlieren images of blast wave evolution from the open end of the shock tube when operated using the two configurations (initial fill pressure of oxyhydrogen is 3 bar in both the cases). The stronger color gradients in the first case indicate the higher strength of the primary and secondary blast waves. When the biological sample is placed in the cavity at the end of the shock tube with the silicone rubber in between, the shockwave energy is transmitted through the silicone membrane to the biological sample. The pressure signals of the transmitted shockwave have been measured in the cavity when the shock tube is operated in the two different configurations (Supplementary Fig. S2a and b). The peak pressure, steady time duration and impulse of the transmitted shockwave have been tabulated (Supplementary Table 1). From the table, it can be seen that the peak pressure and impulse of the shockwave generated is higher for configuration-I while the steady time duration is higher for configuration-II. Experiments carried out to compare the transformation efficiencies of *E.coli* using the two configurations have been shown (Supplementary Fig. S2c). The transformation efficiencies using the shockwave method are significantly higher than the conventional heat shock method and also the results reveal that a higher pulse duration of the shockwave is required for efficient bacterial transformation. Therefore, all the remaining experiments reported in this paper were carried out using configuration-II of the shock tube.

### Optimization of parameters for bacterial transformation

Shockwaves have been reported to show a deleterious effect on the genomic as well as extra chromosomal DNA of bacteria. It was observed that shock waves used for the experiments described do not have a significant effect on the viability of bacteria and integrity of the plasmid DNA. Only *Salmonella* Typhimurium shows a reduced viability,although statistically not significant, at higher fill pressures of the shock tube (Fig. S3).The dependence of transformation efficiency on the total number of bacterial cells used was also analyzed. It was observed that with an increase in the concentration of bacteria the efficiency of plasmid transfer significantly increased (Fig. S3). Different concentrations of calcium chloride ranging from 100 mM to 400 mM were used for transformation. Optimum transformation efficiency was obtained at a concentration of 200 mM CaCl_2_ (Fig. S3). For transformation in *Mycobacteria*, growth medium was supplemented with 1.5% glycine. This has been reported to aid in increasing the bacterial cell wall permeability. Bacterial transformation was also confirmed at the levels of gene expression by examining the bacteria for the expression of the mCherry red protein or green fluorescent protein using a confocal microscope (Fig. S4). Post optimizing the conditions for transformation in *E.coli*, the device was used to carry out transformations in other bacteria *Salmonella* Typhimurium, *Pseudomonas aeruginosa, Mycobacterium smegmatis, Mycobacterium tuberculosis* and *Helicobacter pylori*.

### Effect of fill pressure of oxyhydrogen mixture on the transformation efficiency

**Figure 2:**
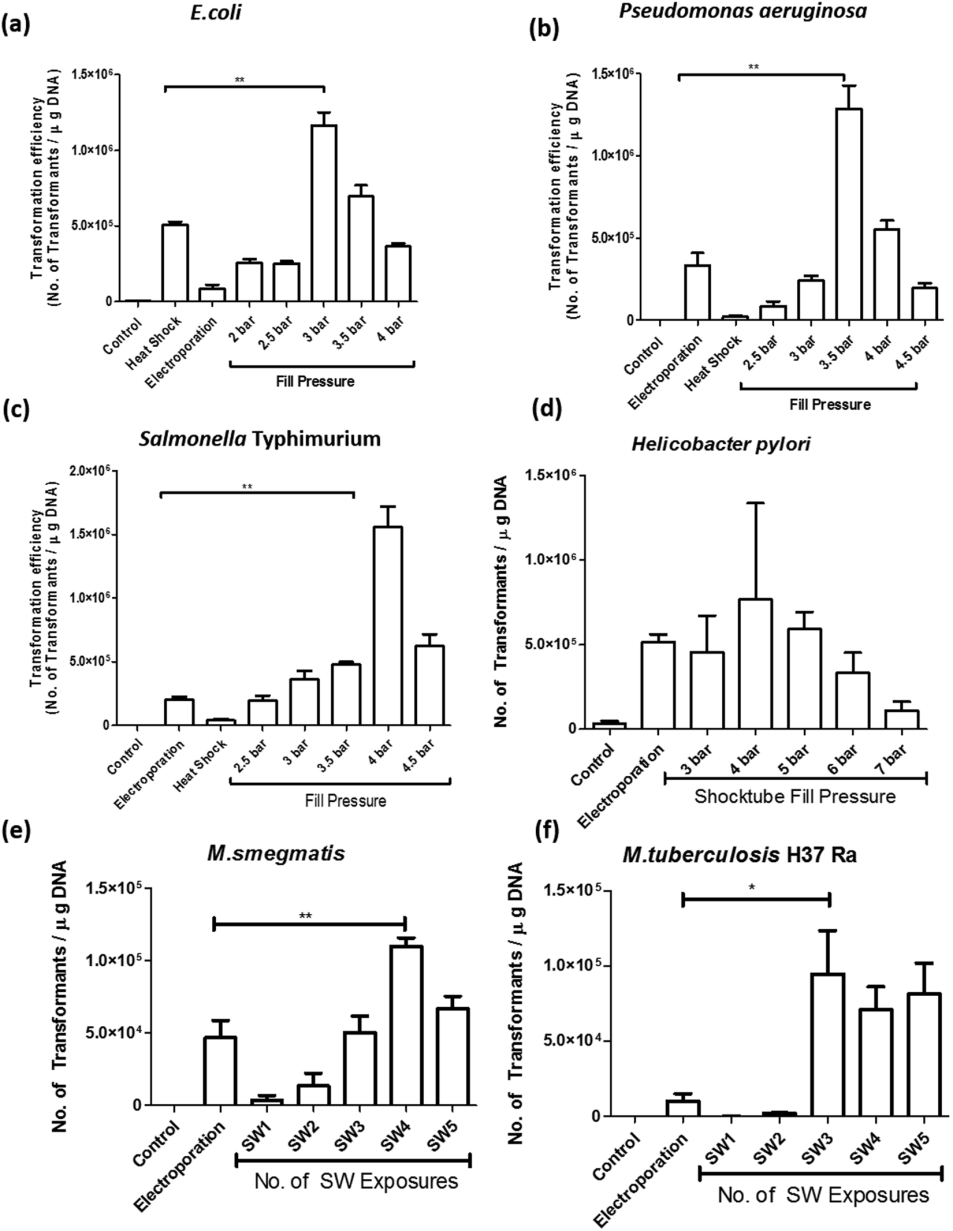
Efficiency of bacterial transformation using shockwaves and its comparison with electroporation. (**a**) *E.coli*, (**b**) *P.aeruginosa*, (**c**) *Salmonella* Typhimurium, (**d**) *H. pylori*, (**e**) *Mycobacterium smegmatis* and (**f**) *Mycobacterium tuberculosis* were transformed with relevant plasmids mainly by heat shock, electroporation and shock waves. After transformation LB broth was added to the cells, incubated at 37 °C for 1 h before plating. For *E.coli, P.aeruginosa*, *Salmonella* and *H.pylori* the shocktube fill pressure was varied. For Mycobacterial transformations, the shocktube fill pressure was maintained at 10 bar and the number of consecutive shockwave exposures were varied to achieve high efficiency transformation. Post incubation, CFU were counted and transformation efficiency was calculated. Statistical significance was defined as follows (*P < 0.05, **P < 0.005, ***P < 0.0005) (Student’s t test).

**Figure 3:**
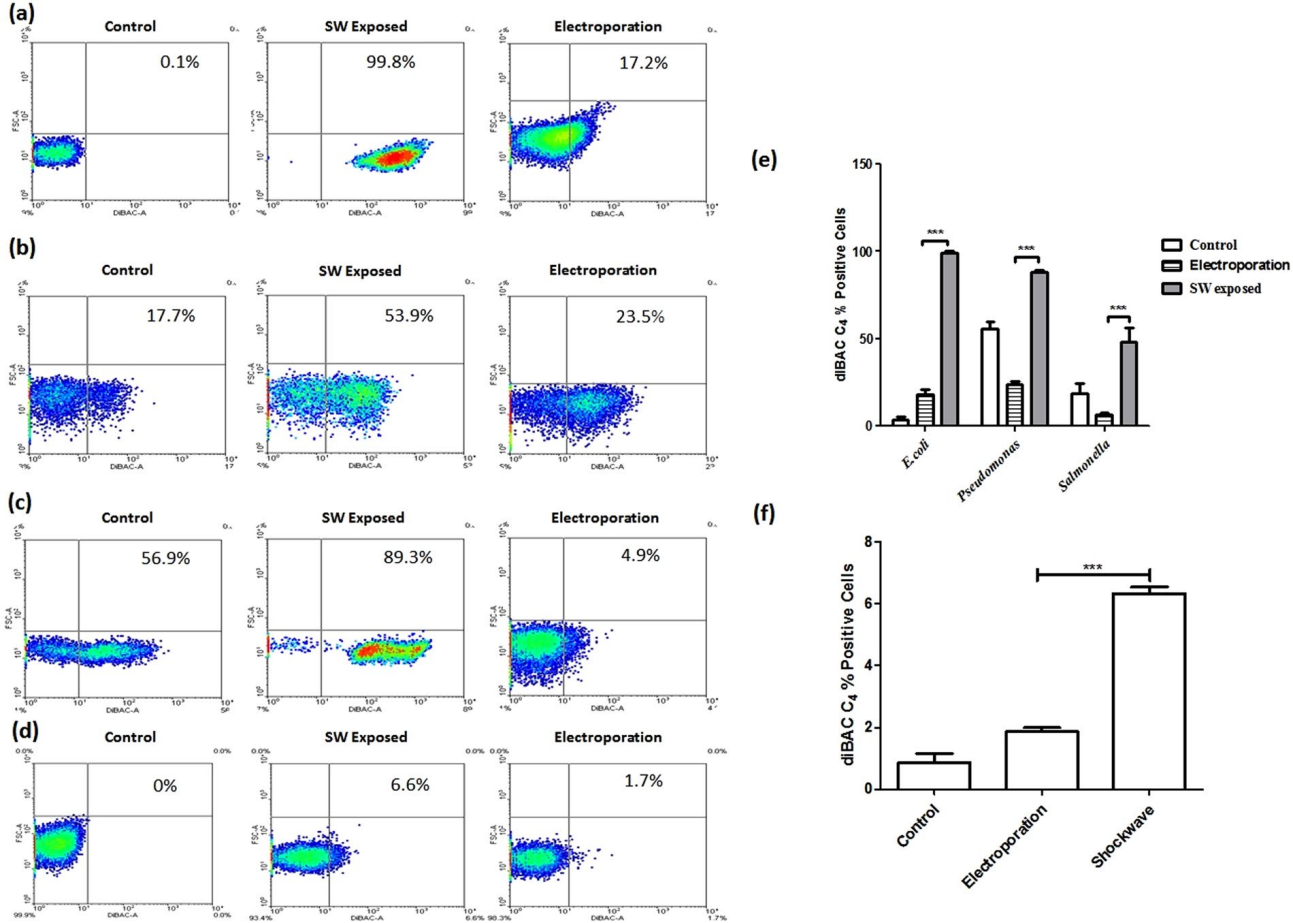
Effect of shockwaves on membrane depolarization. (**a–d**) diBACC4 assay was performed to assess the change in the membrane polarity during the exposure to shockwave in *E.coli, Pseudomonas*, *Salmonella* and *Mycobacterium smegmatis*. The bacteria were incubated with diBACC4 (1 µg/ml) prior to shockwave exposure. The bacteria were analyzed using flow cytometry for change in the membrane polarity. Results are representative of three independent experiments with similar results. (**e–f**) Comparison of the percent diBACC4 positive cells in *E.coli, Pseudomonas*, *Salmonella* and *Mycobacterium smegmatis*. Statistical significance was defined as follows (*P < 0.05, **P < 0.005, ***P < 0.0005) (Student’s t test).

Transformation in *E.coli*, *Pseudomonas aeruginosa*, *Salmonella* Typhimurium*, Mycobacterium smegmatis, Mycobacterium tuberculosis* and *Helicobacter pylori* was performed at different fill pressures of oxyhydrogen to achieve maximum transformation efficiency (Fig. 2a–f). *E.coli* required the least fill pressure for transformation while *Mycobacteria* required the highest fill pressure as well as shockwave pulses amongst the group of bacteria transformed. *Mycobacteria* were exposed to multiple shock wave pulses ranging from 1 to 5 pulses. *Salmonella, Pseudomonas* and *Helicobacter* species required intermediate shock tube fill pressures. It was observed that for every bacterial species, the conditions required for optimal transformation efficiency were different and transformation efficiencies were significantly higher than the conventional method of electroporation. The transient change in the membrane polarity was confirmed by the diBACC_4_ assay in the bacteria when exposed to shockwaves (Fig. 3a–d). Quantification of the membrane depolarization showed significant change after bacteria were exposed to shockwaves (Fig. 3e,f).

### Shockwaves induce increase in bacterial length and Young’s modulus facilitating high efficiency transformation

**Figure 4:**
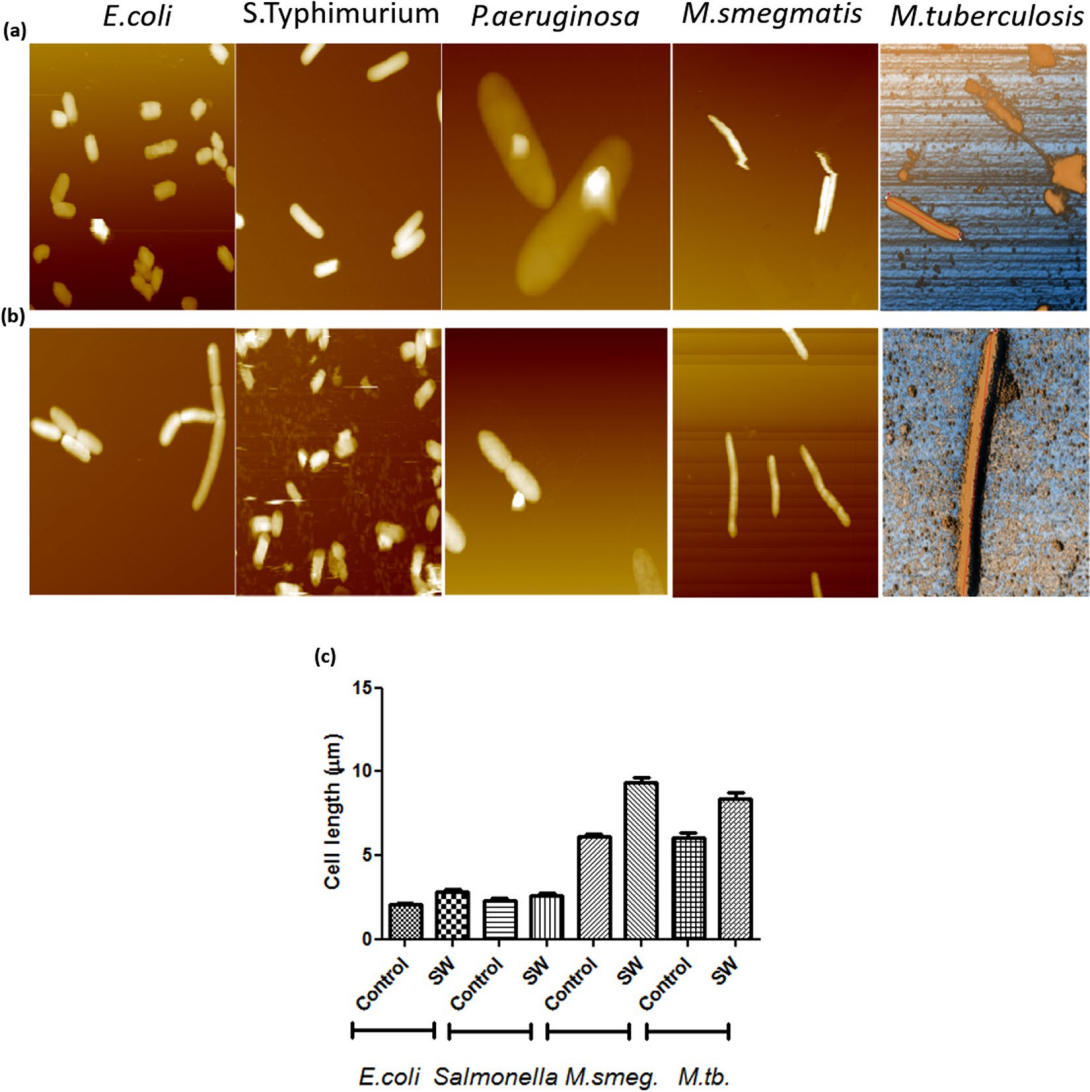
Topographical images of various bacterial species using Atomic force microscopy. (**a**) Untreated bacteria, (**b**) Shockwave treated bacteria. Bacteria were either exposed to shockwaves or were unexposed and were processed for imaging using noncontact mode AFM. Single bacteria were imaged and topographical analysis was done using Park systems software. A total of 10 fields were imaged and analyzed. The results are representative of the measurements. (**c**) Bacterial cell length in control and shockwave treated bacteria was measured and compared. A minimum of 200 bacteria per sample were analyzed.

**Figure 5:**
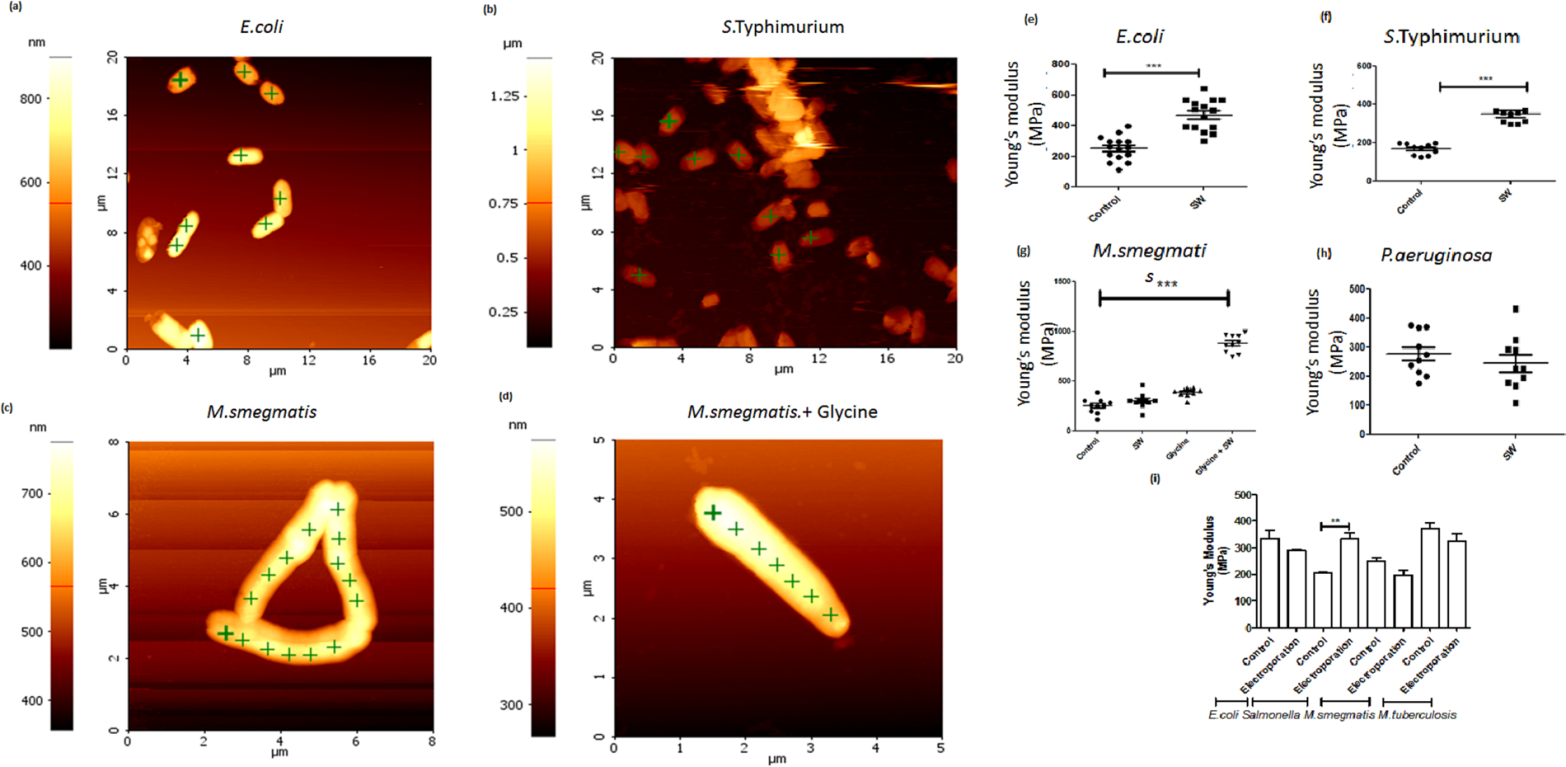
Determination of Young’s modulus of bacteria after shockwave exposure. (**a**) *E.coli*, (**b**) *Salmonella*, (**c**) *M. smegmatis* and (**d**) *M. smegmatis* grown in presence of 1.5% Glycine were either exposed to shockwaves or were unexposed prior to atomic force microscopy for measuring the Young’s modulus, a measure of elasticity. Uniformly spaced points for indentation were selected on the bacterial surface and contact mode indentation was performed. The force displacement curves were analyzed using the Hertzian model. A total of 10 bacteria were analyzed per sample. The images are representative of the experiment. (**e–h**) Comparison of Young’s modulus after shockwave exposure. (**i**) Comparison of Young’s modulus after electroporation. Statistical significance was defined as follows (*P < 0.05, **P < 0.005, ***P < 0.0005) (Student’s t test).

Transformation using shockwaves was highly efficient as described earlier but its mechanism was still unclear. To check the effect of shockwaves, bacteria were observed immediately under an atomic force microscope (Fig. 4a,b). Bacterial cell length was quantified in the control and shockwave exposed sample (Fig. 4c). It was observed that on an average, the cell length in the shockwave exposed sample was increased to 1.5-fold as compared to control. This observation was consistent amongst all the bacteria used for transformation wherein Mycobacteria showed the most significant increase in the cell length after shockwave exposure. Length being an important parameter in determining the stiffness and Young’s modulus of any rigid body, the Young’s modulus of bacteria pre and post-exposure to shockwaves was measured (Fig. 5a–d). Force displacement curves were generated. Hertzian model of data fit was used to compute the Young’s modulus. It was observed that upon shockwave exposure, the Young’s modulus increased significantly in all the bacterial species used (Fig. 5e–h). Next, Young’s modulus was also measured after electroporation in various bacteria. It was observed that efficient bacterial transformation was directly related to the change in Young’s modulus (Fig. 5g). In case of less efficient transformation by electroporation in Mycobacteria, it was seen that the change in Young’s modulus was not significant. Therefore, from these observations, it is concluded that a positive change in Young’s modulus of the bacteria is of utmost importance to achieve high efficiency transformation. Mathematical finite element modeling has also shown the increase in length and Young’s modulus after shockwave exposure (Fig. S6).

### Mechanism of enhanced bacterial transformation using the shockwave device

**Figure 6:**
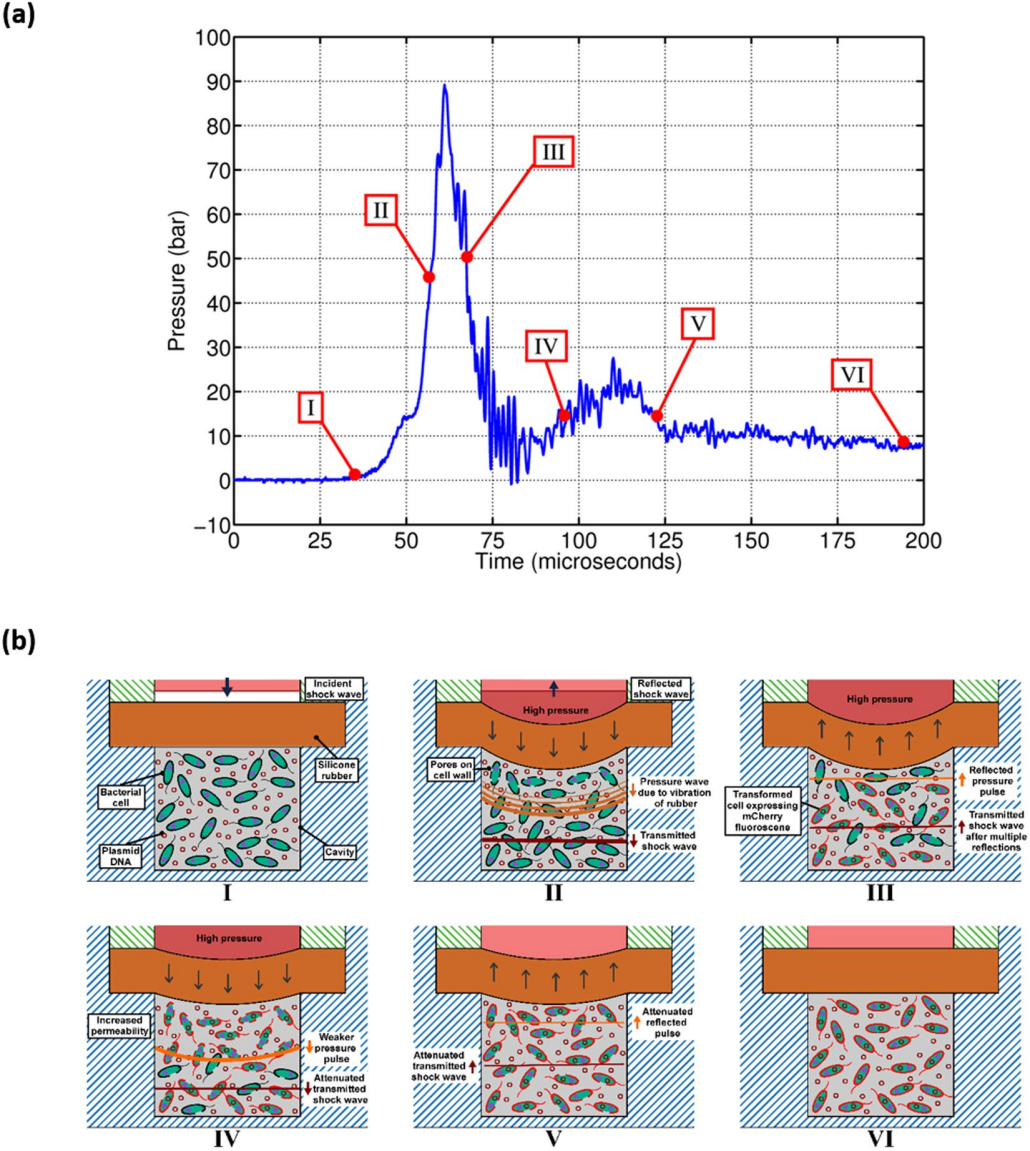
Proposed Mechanism of enhanced bacterial transformation. (**a**) Pressure signal indicating the location of the stages shown in the schematic diagram (Only *E.coli* has been considered) (**b**) Schematic diagram showing the different stages during the bacterial transformation experiment. The pressure pulse due to the vibration of rubber membrane is indicated in orange while the transmitted shock wave is shown in brown.

The incident shock wave, which is the driving force for transformation, undergoes many changes upon hitting the silicone rubber membrane at the end of the shock tube. A major portion of the incident energy is transmitted through the rubber to the bacterial culture in the cavity. A part of the incident energy is absorbed by the rubber membrane while another portion of the energy is reflected back into the shock tube. The pressure measurement inside the cavity gives some useful insight into the possible mechanism of enhanced transformation in the bacteria using the device. Figure 6a shows the pressure experienced by the bacterial culture in the cavity during operation of the device. It can be seen that although the incident shockwave has a pressure jump of only about 7 bar (See Fig. 1d Configuration II), the pressure inside the cavity reaches a pressure of about 90 bar. A closer look at the pressure signal shows the presence of two distinct pressure peaks. The first peak reaches a pressure of about 90 bar and the time period from rise to fall is about 40 microseconds. This time duration can be easily related to the steady time duration of the incident shockwave (~50 microseconds) seen in Fig. 1d Configuration II. The rise in pressure inside the cavity to a pressure of up to 90 bar could be due to the combined effect of the transmitted shockwave and the pressure rise in the liquid due to the bulging of the rubber membrane. The drop in the pressure after the peak is because of the reflected pressure waves from the bottom of the cavity causing the rubber to go back to its original shape and hence creating a pressure relieving effect in the cavity. In Fig. 6a, it can be seen that the second peak reaches up to a pressure of about 28 bar and the time period is about 30 microseconds. Although, this time duration corresponds to a continuous drop in the pressure of the incident shockwave after the steady time, a second pressure peak is still observed in the cavity. This could be because of the fact that the high pressure in the shock tube is still persistent during this time duration but the pressure value is lower in amplitude as compared to the initial pressure jump. Therefore, the rubber bulges again to give the second pressure peak of lower amplitude in the cavity as compared to the first pressure peak.

Also, some high frequency oscillations can be observed in the fall time duration after the first pressure peak. These high frequency oscillations slowly dampen out with time. The vibration of the silicone membrane or the multiple reflections in the cavity could be a reason for these oscillations. The time period for silicone rubber vibrating at its natural frequency lies in the range of 1.2–6.2 milliseconds (see Supplementary Note S1). Therefore, the high frequency oscillations cannot be due to the vibrations of silicone rubber. A simple calculation reveals that the time taken for stress waves to travel inside the cavity is about 3.33 microseconds (see Supplementary Note S2), which is of the same order as the time period of the high frequency oscillations observed in the Fig. 6a. Therefore, the transmitted shock wave undergoes a series of reflections against the bottom of the cavity and the face of the rubber membrane causing high frequency oscillations in the pressure inside the cavity and also continuously attenuating in intensity. Keeping all these points in mind and correlating the pressure signals shown in Fig. 1d Configuration II and Fig. 6a, illustrations of the possible mechanism of bacterial transformation using the shockwave assisted device have been shown in Fig. 6b. The corresponding time instants of the illustrations have been indicated in Fig. 6a.

## Discussion

Shock waves have been extensively used in various interdisciplinary arenas of research and their applications in biomedical engineering have been gaining prominence lately^7, 18^. Bacterial transformation is a technique of prime importance in field of genetic engineering where it finds applications from construction of genomic libraries to production of recombinant insulin for treatment of diabetes. The method of introducing a foreign DNA in a host cell using common techniques like electroporation and heat-shock method, is associated with a number of limitations like low transformation efficiency, tedious competent cell preparation and requirement of an extensively salt free medium^11^. Using shockwaves to perform bacterial transformation has attracted the interest of researchers around the globe because shockwaves not only help to improve the transformation efficiencies as compared to conventional methods but also demonstrates the potential to transform some complex bacterial species. *Mycobacteria* have a thick waxy cell wall due to the presence of mycolic acid which makes it one of the most difficult micro-organisms to transform^19, 20^. In this study, we report a new shockwave-assisted technique for high efficiency transformation of a range of bacterial species which include *E.coli, Salmonella* Typhimurium, *Pseudomonas aeruginosa, Helicobacter pylori* as well as *Mycobacteria*.

The generation of shockwaves is a natural phenomenon that is a result of a sudden release of mechanical, chemical, nuclear or electrical energy. Most commonly, shockwaves are produced in laboratory confinements using equipment like pressurized gas bottles or expensive instruments like lasers or harmful detonable mixtures like RDX and TNT^16, 17^. For the present study, we have built a miniature oxyhydrogen-driven shock tube device that utilizes *in-situ* generated oxyhydrogen mixture for generation of shockwaves. This self-supporting device eliminates the need for storage of harmful chemicals and high pressure gases, avoids issues encountered with improper mixing of detonable mixtures, uses a carbon-free combustion method and provides a flexibility to vary the steady time and peak amplitude of the generated shockwave^12^. All these advantages make it a unique facility to carry out safe and controlled shockwave-assisted biomedical studies with good repeatability.

In the proposed device, the length of the driver and driven sections can be changed to vary the shock wave parameters namely steady time duration and peak amplitude. Two shock tube configurations were studied to understand the role played by these shock wave parameters. Pressure measurements and high-speed Schlieren technique^21^ were used to understand the shockwave generated by the two configurations. *E.coli* was used as the model organism to compare the transformation efficiencies obtained using the two shock tube configurations. Experiments reveal that the configuration with a higher steady time gives best efficiencies and also shows that higher peak pressure and higher impulse does not necessarily guarantee higher transformation efficiency. A longer steady time leads to a significant increase in the transformation efficiency. Various parameters like the bacterial cell density, concentration of calcium chloride and plasmid DNA integrity were tested and standardized.

The device has the provision to increase the strength of the shockwave by increasing the initial fill pressure of the oxyhydrogen mixture. This allows operating the device over a wide range of conditions so that different species of bacteria which include *E.coli, Salmonella* Typhimurium, *Pseudomonas aeruginosa, Helicobacter pylori* and *Mycobacteria* can be transformed using this device. It was observed that the initial oxyhydrogen fill pressure required for the highest efficiency transformation in the different bacteria was not the same. This is due to the diversity in the composition and structure of the cell wall for the different bacterial species. The initial oxyhydrogen fill pressures were varied for each bacterial species to find out the highest efficiency. It was observed that the as the initial oxyhydrogen fill pressure is increased, the transformation efficiency increases but after a specific initial oxyhydrogen fill pressure, the transformation efficiency decreases. The optimum initial oxyhydrogen fill pressure is least for *E. coli* while *Mycobacteria* could be transformed by multiple pulses of a high-pressure shockwave. In general, the transformation efficiency achieved by the shockwave-assisted technique was significantly higher than the conventionally used method of electroporation.

In the present work, we have also performed studies to understand the mechanism of bacterial transformation. DNA uptake is a function of the cell wall rigidity and permeability. To access the status of cell wall permeability, DiBac assay was performed which uses a potential sensitive dye. It was found that shockwaves cause a transient depolarization of the bacterial cell wall which could be the cause of DNA uptake. The stiffness of the cell wall was also measured in control as well as shockwave treated bacteria. Shockwave exposure causes a significant increase in Young’s modulus of the bacteria. This observation was consistent over all the bacteria used for transformation. We speculate that this increase in the Young’s modulus is responsible for inducing competence in the bacteria. The pores present on the cell wall enlarge due to shockwave exposure which leads to DNA uptake from the environment. Computational finite element modelling also confirms the experimental observation of bacterial elongation during shockwave mediated transformation (Fig. S5 and Supplementary note S3). The pressure signals in the sample cavity were also analyzed. The pressure the cavity was found to be oscillating for a finite time become dampening. These oscillations from silicone membrane lead to formation of secondary shockwaves in the cavity. We hypothesize that these multiple shockwave exposures increase the DNA uptake and hence are important in enhancing the transformation efficiency. Hence, we report development of a novel shockwave driven method for transformation and propose a mechanism of transformation using the same.

## Methods

### Operation of the oxyhydrogen-driven miniature shock tube

The biological sample is placed in the sterile cavity and clamped at the end of the shock tube. Tracing paper is used as a diaphragm between the driver and driven section of the shock tube. A similar paper diaphragm is placed at the top of the driver section to enable an instantaneous release of the combustion products and to prevent the formation of a reflected shock wave. This mechanism also ensures that the sample is exposed only to the primary shock wave. Air at ambient atmospheric pressure is present in the driven section of the shock tube. The electrolysis process in the oxyhydrogen generator is initiated when the power supply from the DC source is switched on. The oxyhydrogen generator produces stoichiometric ratio of hydrogen and oxygen gases at around 8 ml/s. As soon as the required initial fill pressure in the driver section of the shock tube is reached, the power supply is switched off. By evacuating the gas between the oxyhydrogen generator and the miniature shock tube, it is ensured that the detonation front does not travel into the oxyhydrogen generator when the shock tube is operated. The miniature shock tube is operated by igniting the mixture using a spark plug. For the subsequent run, the ruptured paper diaphragms are replaced with new ones. The biological sample is replaced with a new sample. The schematics shown in Figs 1a–c and 6b have been drawn in house.

### Pressure measurements in the setup

The pressure in the shock tube is measured using a piezoelectric pressure sensor (PCB Piezotronics, USA). This sensor is flushed mounted to the wall of the shock tube. This sensor measures the pressure of the initial shock wave generated at the open end of the driven section without any cavity attached. To measure the head-on pressures experienced by the liquid in the cavity, the sensor is mounted on the base of the cavity. A polyvinyldifluoride (PVDF)-coated needle hydrophone (Dr. Müller Instruments, Germany, Model no. 113B23, resonant frequency ≥500 kHz) is used to measure the pressure experienced by the bacterial culture in the cavity. The needle gauge is placed in the cavity so that the tip of the sensor is in contact with the bacterial culture. All the pressure signals obtained from the sensors are recorded in an oscilloscope (Yokogawa Electric, Japan).

### Strains and Plasmids

*Escherichia coli* DH5α, *Salmonella* Typhimurium, *Pseudomonas aeruginosa*, *Mycobacterium smegmatis, Mycobacterium tuberculosis* H37 Ra and *Helicobacter pylori* were used for the transformation experiments. Plasmids pFPV encoding mCherry (Addgene GenBank ID AY678264) and pBen encoding mycobacterium specific GFP were used for transformation. Ampicillin (100 µg/ml) or Kanamycin (50 µg/ml) was used for selecting the positive transformants as per the case. Plasmid DNA was isolated from stationary phase cultures of *E.coli* using the alkali lysis method (34). The concentration of DNA was measured by using Nanodrop ND-1000 spectrophotometer and agarose gel electrophoresis. All the chemicals unless mentioned otherwise were used from Sigma Aldrich Ltd.

### Media and Reagents

Bacteria were grown in Luria Bertani (LB) broth and Luria Bertani Agar containing either of the following antibiotics for selection of transformants. Ampicillin (100 µg/ml), Kanamycin (50 µg/ml) were used for selection of transformants in *Escherichia coli*, *Salmonella* Typhimurium, *Pseudomonas aeruginosa*, *Mycobacterium smegmatis, Mycobacterium tuberculosis* H37 Ra and *Helicobacter pylori*.

### Viability Assay

200 µl suspension of competent cells of *Escherichia coli*, *Salmonella* Typhimurium, *Pseudomonas aeruginosa*, *Mycobacterium smegmatis, Mycobacterium tuberculosis* H37 Ra and *Helicobacter pylori* were exposed to shockwaves at different fill pressures ranging from 2 bar to 10 bar. Sterile recovery medium was added to the culture. Culture unexposed to the shockwaves served as the control. The cultures were suitably diluted and plated on to either LB agar or Middlebrook 7H10 agar and incubated for the viable colonies to appear. The CFU count was done and plotted using Graph Pad Prism 5 software.

### Integrity of the Plasmid DNA

200 ng of plasmid DNA (pFPV-mCherry) was suspended in 200 mM CaCl2 and 10% glycerol. This mixture was exposed to shockwaves. The mixture was aspirated; the volume was made up to 1 ml with sterile water. The DNA was precipitated using ice-cold isopropanol by centrifugation at 13500 rpm for 15 minutes at 4 °C.The pellet was re-suspended in sterile water and was visualized for the integrity on 1% agarose gel.

### Preparation of competent cells for transformation

It has been shown that shockwave-mediated transformation is independent of the growth phase of the bacteria (20). Therefore, stationary phase cultures of bacteria to be transformed were centrifuged at 6000 rpm for 10 minutes. The pellets were resuspended in 200 mM Calcium chloride and were incubated on ice for 30 mins. The cultures were centrifuged at 6000 rpm for 10 minutes and the pellet fraction was suspended in 200 mM Calcium chloride and 50% Glycerol. These cells were further used for all the transformation experiments. Mycobacteria were grown in presence of 1.5% glycine to improve transformation efficiency.

### Shockwave mediated transformation

An aliquot of 200 µl of competent cells was mixed with 1 ng/ µl plasmid DNA and was incubated on ice for 30 minutes. Post incubation the mixture was placed in the cavity for exposure to shockwaves. The mixture was exposed to shock waves at different shock tube fill pressures ranging from 2 bar to 10 bar for single or multiple times. The cells were immediately aspirated and 800 µl sterile recovery medium (LB) was added. The cells were incubated at 37 °C for 1 h for *Escherichia coli*, *Salmonella* Typhimurium and *Pseudomonas aeruginosa* for 3 h for *Mycobacterium smegmatis, Mycobacterium tuberculosis* H37 Ra and *Helicobacter pylori* under shaking conditions for the expression of the antibiotic resistance gene. The transformants were selected by plating the cells on antibiotic containing medium.

### Confirmation of Transformation

20–25 colonies were randomly picked from the antibiotic containing LB agar plate. The putative transformants were grown in liquid LB medium for 10–12 h at 37 °C. The plasmids were isolated from these cultures for further validation. The expression of the reporter gene (mCherry/GFP) was also confirmed using confocal microscopy.

### Membrane Depolarization Assay

Membrane depolarization was assessed by using diBACC_4_, a potential sensitive dye. Bacteria were incubated with diBACC4 (1 µg/ml) prior to shockwave exposure. Post exposure, bacteria were washed by centrifugation to remove excess dye and were resuspended in sterile PBS. Analysis was done using flow cytometry for change in the membrane polarity.

### Topology and Cell Wall stiffness measurements

Bacterial samples were diluted 1:100 with sterile deionized water immediately after shockwave exposure. 50 µL of the bacterial suspension was spotted on a glass coverslip and was allowed to air dry in sterile conditions. The coverslip was washed to remove any unbound bacteria and was air dried. The coverslips were then fixed onto a magnetic stub using double-sided carbon tape and transferred to the AFM stage for imaging. AFM measurements were performed as follows. A high-force-constant (∼40 N m^− 1^) silicon AFM probe tip (Acta; Park Systems) was used with a resonating frequency of 300 kHz. The AFM instrument (NX-10 Park Systems) was operated in the non-contact mode. The cantilever was 125 μm in length, 35 μm in width and 4.5 μm in thickness. The tip shape was pyramidal with a tip radius < 10 μm.

### Statistical Analysis

Statistical analysis was performed using Graph pad prism 5. Student’s T test and an unpaired post T test was done unless mentioned otherwise. Significance was considered as follows *P < 0.05, **P < 0.005, ***P < 0.0005.

## Electronic supplementary material

Supplementary Data
