# Supplementary material for: Mechanism of transformation in Mycobacteria using a novel shockwave assisted technique driven by in-situ generated oxyhydrogen
Source: Sci Rep. 2017 Aug 17;7:8645. doi: 10.1038/s41598-017-08542-5 (PMC5561244; doi:10.1038/s41598-017-08542-5)
Supplement: Supplementary file 1 — Supplementary Data [file 41598_2017_8542_MOESM1_ESM.pdf]

Mechanism of transformation in *Mycobacteria* using a novel shockwave assisted technique driven by in-situ generated oxyhydrogen

Akshay Datey[1,2,3,#], Janardhanraj Subburaj[1,#], Jagadeesh Gopalan[1,3]* and Dipshikha Chakravortty[2,3]*,

[1]*Department of Aerospace Engineering, Indian Institute of Science, Bangalore, India.*

[2]*Department of Microbiology and Cell Biology, Indian Institute of Science, Bangalore, India.*

[3]*Centre for Biosystems Science and Engineering, Indian Institute of Science, Bangalore, India.*

SUPPLEMENTARY FIGURES & INFORMATION

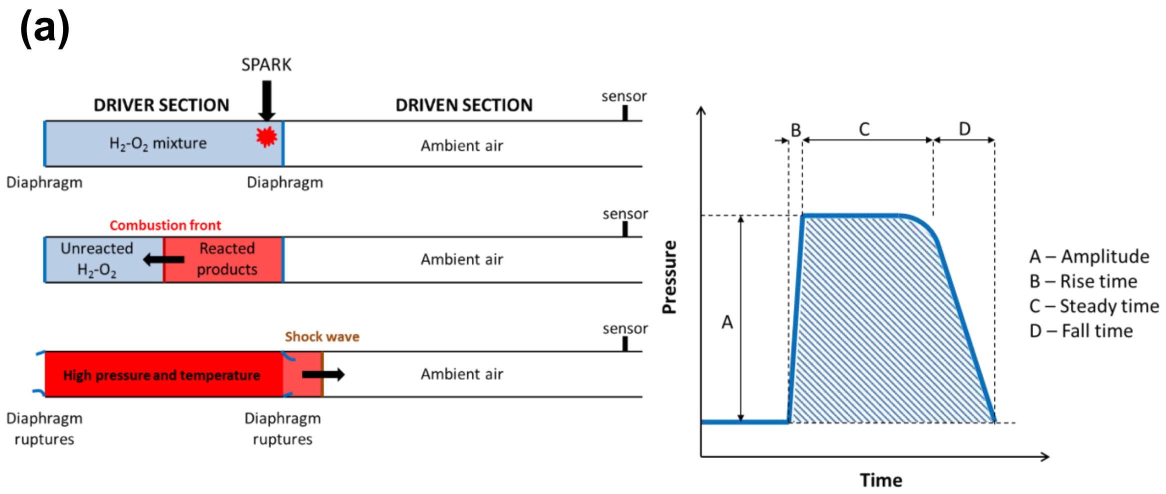

Figure-S1. Working principle of the oxyhydrogen detonation-driven miniature shock tube.

(a) A pictorial representation of the different stages of working of an oxyhydrogen combustion driven shock tube. **(b)** The typical pressure signal of a shock wave generated as measured by a pressure sensor located at the end of the driven section. The shaded region represents the impulse generated by the shock wave.

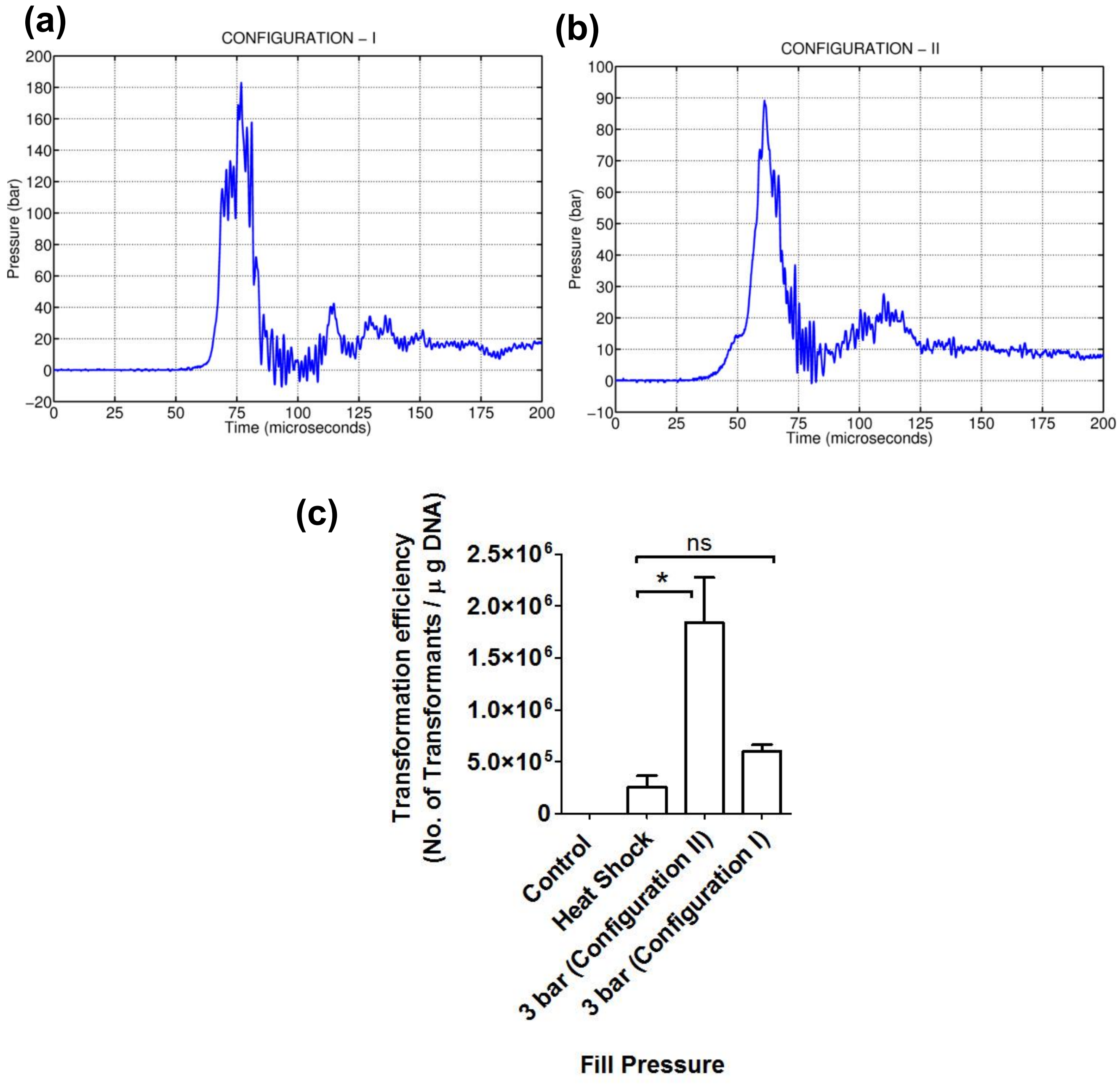

Figure-S2: Effect of steady time and amplitude of the shock wave on the pressure experienced by the bacterial strain (*Escherichia coli*) in the cavity.

(a) Head-on pressure signal measured at the base of the cavity. The device is operated using Configuration-I and oxyhydrogen initial fill pressure of 3 bar. **(b)** The pressure signal for device operated at the same oxyhydrogen initial fill pressure of 3 bar but using Configuration-II. **(c)** Comparison of transformation efficiency in *Escherichia coli* using Configuration-I and Configuration-II (initial fill pressure of oxyhydrogen is 3 bar for both cases).

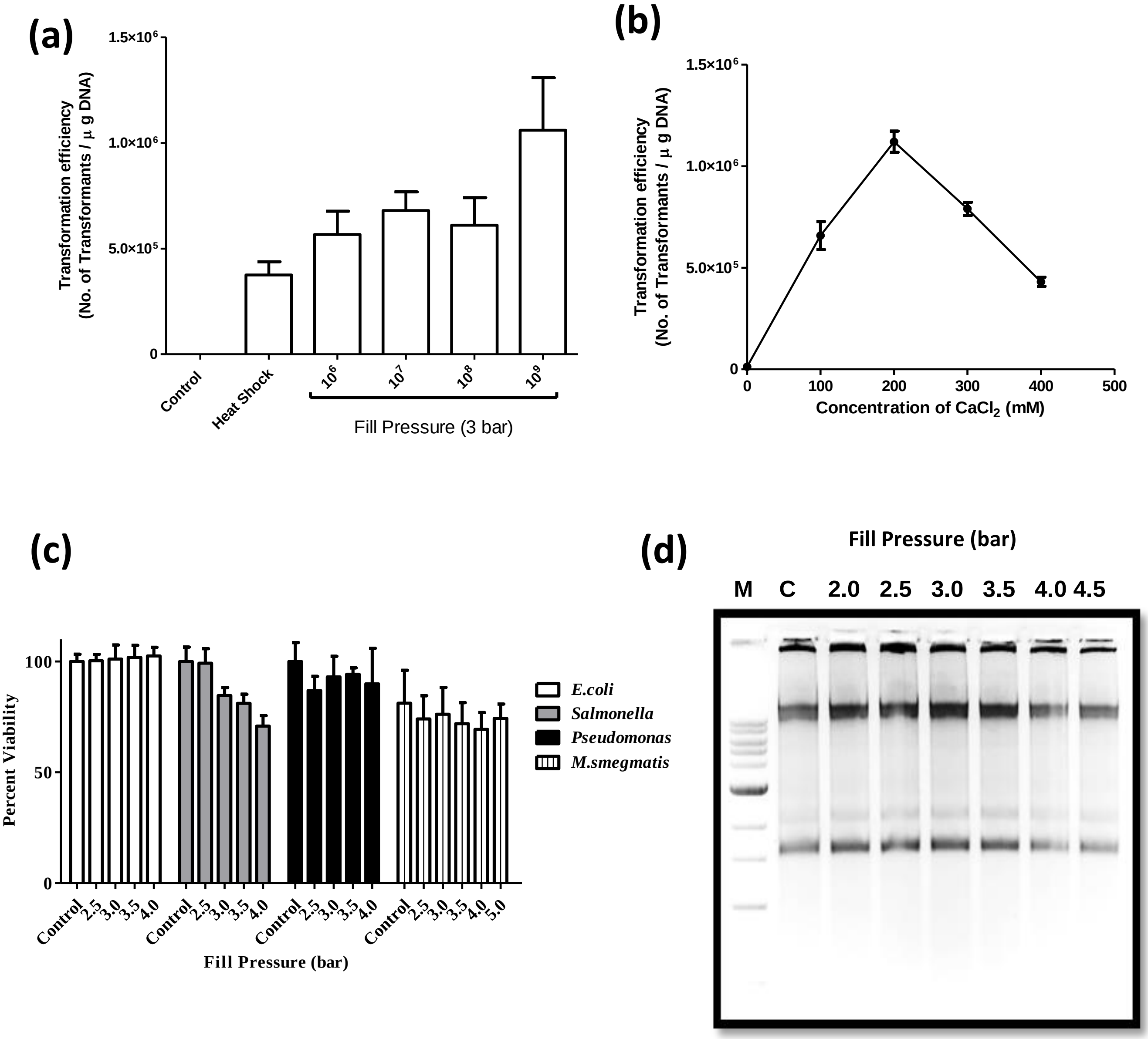

FigureS3: Optimization of parameters for efficient transformation.

(a, b) Different concentrations of bacterial cells (*E.coli*) and $CaCl_2$ were used to evaluate their effect on transformation efficiency. The chemical parameters were optimized enhanced transformation efficiency and **(c)** Bacterial viability was evaluated after shockwave exposure. **(d)** Plasmid DNA was exposed to shockwaves at different shock tube fill pressures and integrity was checked on 1% agarose gel. M and C refer to 1 kb DNA ladder and Control DNA respectively.

(a)

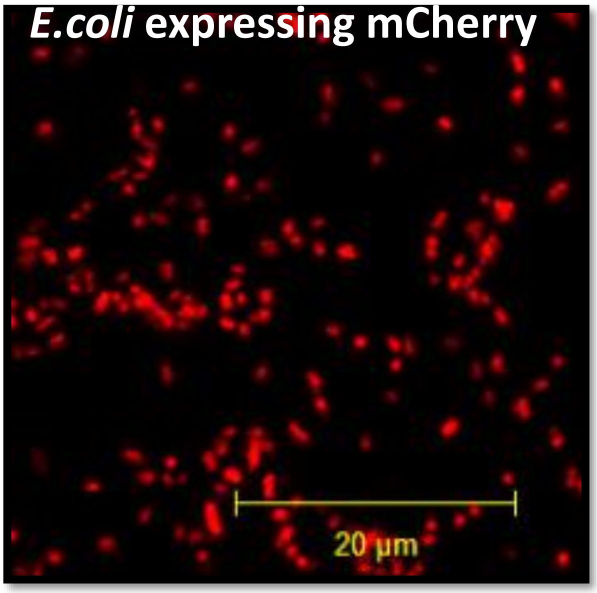

(b)

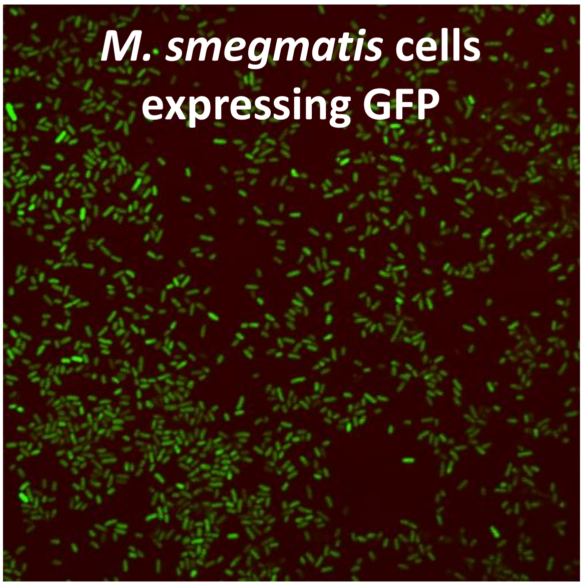

(c)

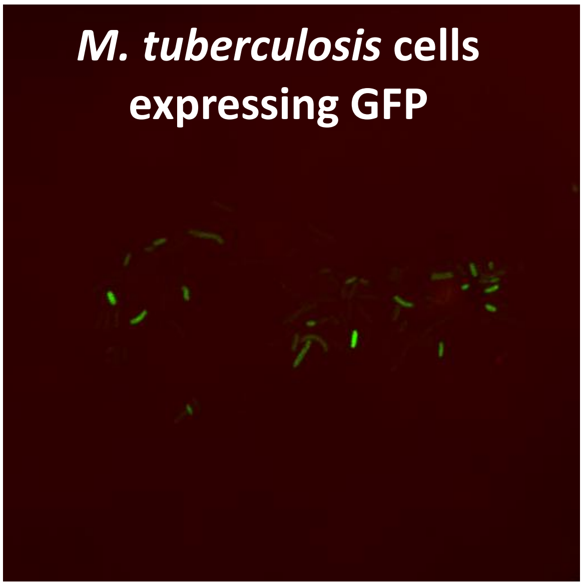

Figure S4: Confirmation of bacterial transformation using fluorescence microscopy.

(a, b, c) *E.coli, M. smegmatis* and *M.tuberculosis* expressing the marker protein namely mCherry and GFP respectively confirming the transformation.

Figure S5

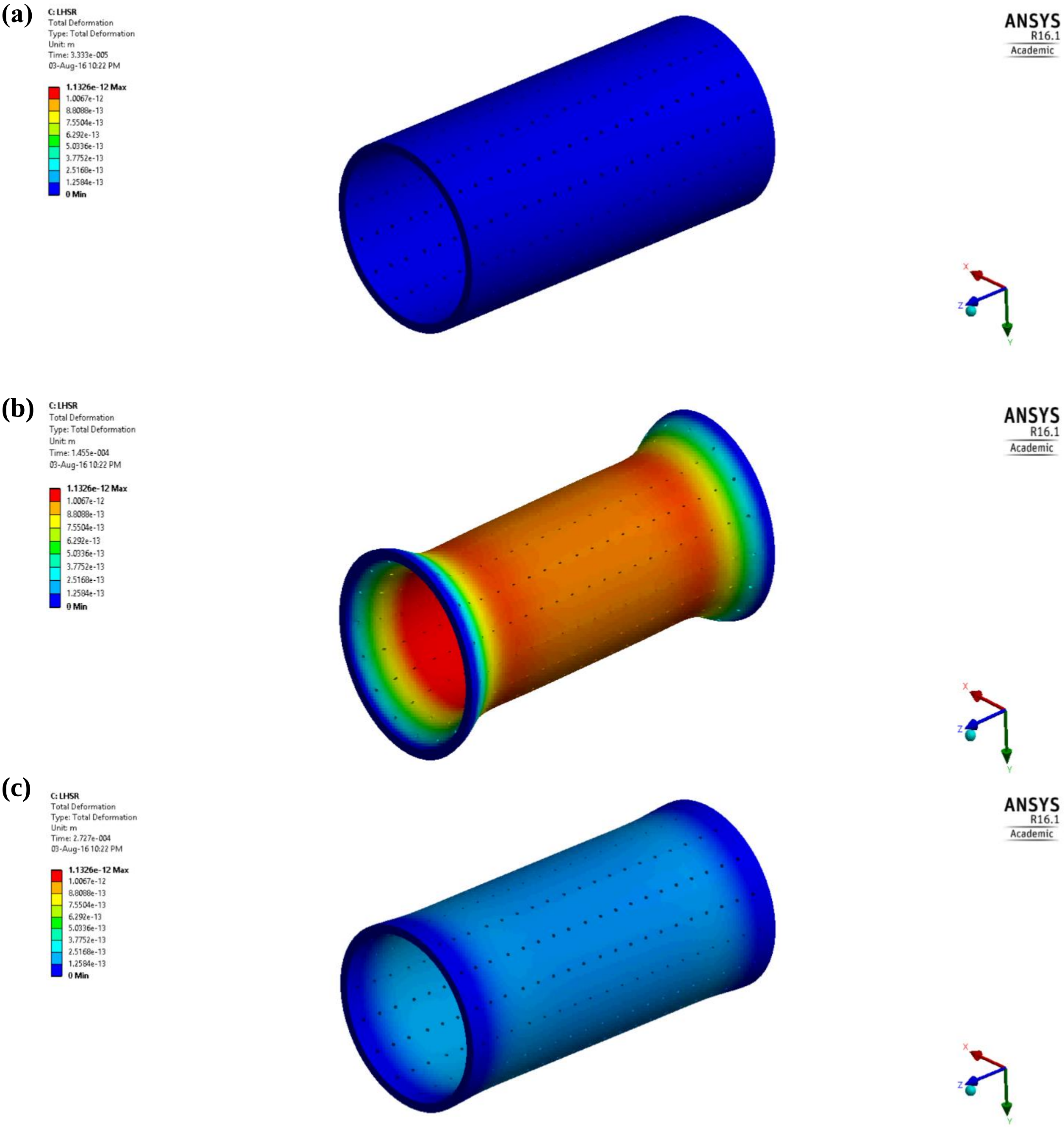

Figure S5: Modelling the topological changes during shockwave mediated transformation.

(a) Model of the bacterial cell wall at rest (t=0), (b) Topography of the bacterial cell wall after shock loading and (c) Bacterial cell wall after shockwave exposure.

Shock tube parameters		Shock wave parameters		
Configuration	Fill pressure (bar)	Peak pressure (bar)	Pulse duration (μs)	Impulse (Pa. s)
I	3.0	183	31.9	214.6
II	3.0	89	48.6	122.1

Supplementary Table-I: Calculated values of shock wave parameters for the pressure signals shown in Supplementary Figure S2a and S2b.

Supplementary Video 1: Schlieren video of the blast evolution from the open end of the device when operated using configuration-I. The initial oxyhydrogen fill pressure is 3 bars.

Supplementary Video 2: Schlieren video of the blast evolution from the open end of the device when operated using configuration-II. The initial oxyhydrogen fill pressure is 3 bars.

SUPPLEMENTARY NOTES

NOTE S1: Estimation of natural frequency of silicone rubber clamped at the edges

The angular natural frequency (ω_n) for a circular plate clamped at the edges is given by[1],

Equation 1:

$$\omega_n(in\, rad/s) = B\sqrt{\frac{Et^3}{\rho a^4(1-\vartheta^2)}}$$

where,

E – Young's modulus (Pa)
t – Thickness of plate (m)
ρ – Density of material (kg/m^3)
a– Diameter of plate (m)
ϑ – Poisson's ratio

The value of B for the first mode of vibration is 11.84. The thickness of the silicone rubber used for the present work is 2mm while the exposed diameter of the silicone rubber is 6mm which is same as the inner diameter of the shock tube. Since, the exact values of the mechanical properties of the silicone rubber used is not known, a range of values for the mechanical properties of silicone rubber is used for calculations and is tabulated below[2].

	**Maximum**	**Minimum**
$\boldsymbol{E}$	50 MPa	1 MPa
$\boldsymbol{\rho}$	2300 kg/m^3	1100 kg/m^3
$\boldsymbol{\vartheta}$	0.49	0.47

For Maximum case,

$$\omega_n = B\sqrt{\frac{Et^3}{\rho a^4(1-\vartheta^2)}} = 11.84 \times \sqrt{\frac{50 \times 10^6 \times (0.002)^3}{2300 \times (0.006)^4 \times (1-0.49^2)}} = 4975\, rad/s$$

For Minimum case,

$$\omega_n = B\sqrt{\frac{Et^3}{\rho a^4(1-\vartheta^2)}} = 11.84 \times \sqrt{\frac{1 \times 10^6 \times (0.002)^3}{1100 \times (0.006)^4 \times (1-0.47^2)}} = 1005\, rad/s$$

Substituting the corresponding values in equation 1, the value of 'ω_n' for the maximum and minimum values of mechanical properties is found to be 4975 rad/s and 1005 rad/s respectively. The natural frequency $\left(f = {}^{\omega_n}/_{2\pi}\right)$ corresponding to these values are 791 Hz and 160 Hz respectively. Therefore, the time period ($t = {}^{1}/_{f}$) corresponding to the natural frequency is 1263 μs and 6250 μs respectively.

References:

[1] Harris CM, Piersol AG. *Harris' shock and vibration handbook. Sixth Edition.* New York: McGraw-Hill; (2010).

[2] http://www.azom.com/properties.aspx?ArticleID=920

NOTE S2: Estimation of time taken by stress waves to travel along liquid column

The speed of sound in a medium is given by,

Equation 2

$$c = \sqrt{\frac{K}{\rho}}$$

Where,

K – Bulk modulus of the medium

ρ – Density of the medium (considered close to density of water)

Hence, the speed of sound in water is approximately 1500 m/s. Therefore, time taken by acoustic wave to travel in a liquid column of height 5mm is 3.33μs.

NOTE S3: Transient Structural Analysis of Bacterial Cell Subjected to Shock Loading

The bacterial cell has been modelled as a hollow cylinder with radius, wall thickness and length being 0.5 μm, 100 nm and 2 μm, respectively. In order to simulate the pores present in the bacterial wall, a uniform distribution of holes having 10 nm as radius is modelled. However, the hemispherical caps of the bacterial cell are not modelled. The bacterial cell model is assigned with experimentally measured material properties which are measured prior to shock exposure. The properties being: Mass of 1 picogram, volume of 0.6 μm^3 and Elastic Modulus of 160 MPa. A Poisson ratio of 0.49 is used[1]. The geometric model is meshed using higher order 3D 20 noded solid elements having translational and rotational degrees of freedom (dof). The mesh density is increased in the vicinity of the pores to accurately capture the deformation behavior. The flat faces of the cylindrical geometry are assumed to be fixed in all dof. The loading to the geometry involves applying transient pressure load generated from experimental shock loading data, normal to the external cylindrical surface of the meshed model. Internal pressure of 1 bar is applied radially outward to the inner cylindrical surface to simulate the bacterial fluid turgor pressure. With the total analysis time being 300 μs, time steps as low as 1 μs is used to solve the transient problem. An iterative solver using Newmark time integration method is utilized for solving the following basic equation of motion,

$$[M]\{\ddot{x}\} + [C]\{\dot{x}\} + [K]\{x\} = [\{F(t)\}]$$

Where, [M] = mass matrix, [C] = damping matrix, [K] = stiffness matrix, {x} = nodal displacement vector (• and •• represents first and second derivative i.e. velocity and acceleration, respectively) and {F(t)} = load vector.

The solved model is post processed to primarily obtain the total deflection of model as well as directional deflection of the pores in order to assess the shock induced elongation of the bacterial cell and pore in the cell wall.

Reference

[1] L.Zhao, D.Schaefer, H.Xu, S. J. Modi, W. R. LaCourse, M. R. Marten*, *Elastic Properties of the Cell Wall of Aspergillus nidulans Studied with Atomic Force Microscopy*, Biotechnol. Prog. 2005, 21, 292-299.
